# Tenulin 0.25-hydrate, a sesquiterpene lactone isolated from *Helenium amarum*

**DOI:** 10.1107/S1600536813018369

**Published:** 2013-07-10

**Authors:** Kyle S. Knight, Cole T. Smith, Thomas G. Waddell, Bruce Noll

**Affiliations:** a Department of Chemistry, The University of Tennessee at Chattanooga, Chattanooga, TN 37403, USA; b Crystallographic Systems, Bruker AXS Inc., 4565 East Cheryl Parkway, Madison, WI 53711, USA

## Abstract

The asymmetric unit of the title compound, C_17_H_22_O_5_·0.25H_2_O [systematic name: 2-hy­droxy-2,2a,6,9a-tetra­methyl-2a,4a,5,6,6a,9a,9b,9c-octa­hydro-2*H*-1,4-dioxadi­cyclo­pent[*cd*,*f*]azulene-3,9-dione 0.25-hydrate], a natural product isolated from *Helenium amarum*, contains two independent tenulin mol­ecules and half a water mol­ecule of crystallization situated on a twofold rotation axis. The hy­droxy group of the hemiketal moiety is in a β-position. In the crystal, each water mol­ecule inter­acts with four tenulin mol­ecules *via* O—H⋯O hydrogen bonds. The two independent tenulin mol­ecules (*A* and *B*) differ only in the character of their participation in hydrogen bonding. Specifically, while *A* is an acceptor of O_water_—H⋯O_*A*_ and a donor of O_*A*_—H⋯O_*B*_ hydrogen bonds, mol­ecule *B* is an acceptor of the latter hydrogen bond and the donor of an O_*B*_—H⋯O_water_ hydrogen bond. In the crystal, these O—H⋯O hydrogen bonds link the tenulin and water mol­ecules into layers parallel to the *ac* plane.

## Related literature

For the discovery and structural identification of tenulin, see: Clark (1939 ▶); Herz & Sharma (1975 ▶); Braun *et al.* (1956 ▶); Barton & De Mayo (1956 ▶). For the biological activity of tenulin and its analogs, see: Lee *et al.* (1977 ▶); Waddell *et al.* (1979 ▶); Hwang *et al.* (1996 ▶); Li & Zhang (2008 ▶); Hodge & Waddell (1995 ▶) and references therein. For the crystal structure of bromo­isotenulin, see: Mazhar *et al.* (1974 ▶).
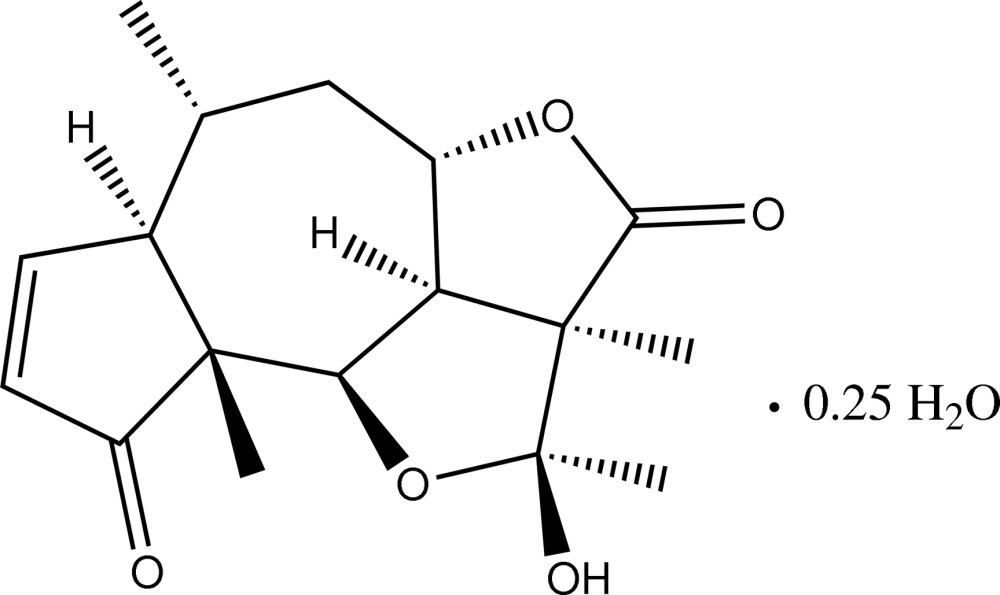


## Experimental

### 

#### Crystal data

C_17_H_22_O_5_·0.25H_2_O
*M* *_r_* = 310.86
Orthorhombic, 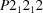

*a* = 10.5508 (16) Å
*b* = 28.371 (4) Å
*c* = 10.5228 (14) Å
*V* = 3149.9 (8) Å^3^
*Z* = 8
Mo *K*α radiation
μ = 0.10 mm^−1^
*T* = 200 K
0.42 × 0.32 × 0.26 mm

#### Data collection

Bruker APEXII CCD diffractometer
Absorption correction: multi-scan (*SADABS*; Bruker, 2008 ▶) *T*_min_ = 0.818, *T*_max_ = 1.000
19558 measured reflections
5543 independent reflections
4678 reflections with *I* > 2σ(*I*)
*R*_int_ = 0.041

#### Refinement

*R*[*F* ^2^ > 2σ(*F* ^2^)] = 0.049
*wR*(*F* ^2^) = 0.121
*S* = 1.26
5543 reflections
415 parameters
1 restraint
H-atom parameters constrained
Δρ_max_ = 0.32 e Å^−3^
Δρ_min_ = −0.22 e Å^−3^

### 

Data collection: *APEX2* (Bruker, 2009 ▶); cell refinement: *SAINT* (Bruker, 2009 ▶); data reduction: *SAINT*; program(s) used to solve structure: *SHELXS97* (Sheldrick, 2008 ▶); program(s) used to refine structure: *SHELXL97* (Sheldrick, 2008 ▶); molecular graphics: *OLEX2* (Dolomanov *et al.* 2009 ▶); software used to prepare material for publication: *OLEX2*.

## Supplementary Material

Crystal structure: contains datablock(s) global, I. DOI: 10.1107/S1600536813018369/cv5419sup1.cif

Structure factors: contains datablock(s) I. DOI: 10.1107/S1600536813018369/cv5419Isup2.hkl

Supplementary material file. DOI: 10.1107/S1600536813018369/cv5419Isup3.cdx

Additional supplementary materials:  crystallographic information; 3D view; checkCIF report

## Figures and Tables

**Table 1:** Hydrogen-bond geometry (Å, °)

*D*—H⋯*A*	*D*—H	H⋯*A*	*D*⋯*A*	*D*—H⋯*A*
O3—H3⋯O10^i^	0.84	1.87	2.711 (3)	175
O8—H8⋯O1*W* ^ii^	0.84	2.17	2.945 (5)	153
O1*W*—H1*W*⋯O5	0.99 (3)	1.94 (3)	2.897 (4)	162 (6)

Symmetry codes: (i) 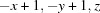; (ii) 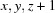.

**Fig. 1.**
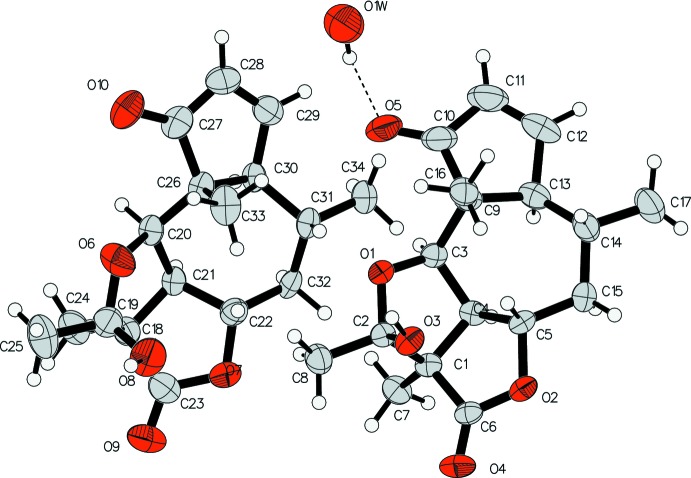
The content of asymmetric unit of the title compound showing the atomic numbering and 50% probablility displacement ellipsoids.

## supplementary crystallographic information

### Comment

Tenulin, C_17_H_22_O_5_, is a sesquiterpene lactone isolated from sneezeweed *Helenium amarum*, a medicinal plant native to the southeastern USA. The crystal structure contains two crystallographically independent tenulin molecules with similar bond dimensions. There is a single molecule of water for every four molecules of tenulin. Each tenulin molecule is linked *via* hydrogen bonding to the central water molecule. Additionally, there is hydrogen bonding between the carbonyl oxygen (O5) of the ketone and the free hydroxy of the hemiketal (O8).

The structure clearly shows the hydroxy of the hemiketal to be in the *beta* position, a point of contention in older structural studies, using only nuclear magnetic resonance. The absolute configuration of all other stereocenters is that established for other sesquiterpene lactones (Mazhar *et al.*, 1974).

### Experimental

tenulin was isolated as described previously (Waddell *et al.*, 1979).

### Refinement

H atoms for the water molecule were found in the difference map and were constrained to ride on the oxygen at a fixed distance of 0.9 Å with *U*_iso_(H)= 1.5Ueq (C). All other H atoms were positioned geometrically, with bond distances of 0.98, 0.99, 1.00 and 0.95 Å for methyl, methylene, methine and vinyl, respectively. All carbon bound H atoms were constrained to ride on their parent atoms with *U*_iso_(H)= 1.2*U*_eq_ (C) or 1.5*U*_eq_ (C) for methyl H atoms.

### Crystal data

**Table:**

C_17_H_22_O_5_·0.25H_2_O	*D*_x_ = 1.311 Mg m^−^^3^
*M**_r_* = 310.86	Mo *K*α radiation, λ = 0.71073 Å
Orthorhombic, *P*2_1_2_1_2	Cell parameters from 6528 reflections
*a* = 10.5508 (16) Å	θ = 2.4–24.7°
*b* = 28.371 (4) Å	µ = 0.10 mm^−^^1^
*c* = 10.5228 (14) Å	*T* = 200 K
*V* = 3149.9 (8) Å^3^	Plate, colorless
*Z* = 8	0.42 × 0.32 × 0.26 mm
*F*(000) = 1332	

### Data collection

**Table:**

Bruker APEXII CCD diffractometer	4678 reflections with *I* > 2σ(*I*)
Graphite monochromator	*R*_int_ = 0.041
φ and ω scans	θ_max_ = 25.0°, θ_min_ = 2.4°
Absorption correction: multi-scan (*SADABS*; Bruker, 2008)	*h* = −11→12
*T*_min_ = 0.818, *T*_max_ = 1.000	*k* = −33→33
19558 measured reflections	*l* = −12→12
5543 independent reflections	

### Refinement

**Table:**

Refinement on *F*^2^	Primary atom site location: iterative
Least-squares matrix: full	Hydrogen site location: mixed
*R*[*F*^2^ > 2σ(*F*^2^)] = 0.049	H-atom parameters constrained
*wR*(*F*^2^) = 0.121	*w* = 1/[σ^2^(*F*_o_^2^) + (0.0607*P*)^2^] where *P* = (*F*_o_^2^ + 2*F*_c_^2^)/3
*S* = 1.26	(Δ/σ)_max_ < 0.001
5543 reflections	Δρ_max_ = 0.32 e Å^−^^3^
415 parameters	Δρ_min_ = −0.22 e Å^−^^3^
1 restraint	

### Special details

**Table:**

Experimental. SADABS-2008/1 (Bruker,2008) was used for absorption correction. wR2(int) was 0.1805 before and 0.0485 after correction. The Ratio of minimum to maximum transmission is 0.8179. The λ/2 correction factor is 0.0000.
Geometry. All e.s.d.'s (except the e.s.d. in the dihedral angle between two l.s. planes) are estimated using the full covariance matrix. The cell e.s.d.'s are taken into account individually in the estimation of e.s.d.'s in distances, angles and torsion angles; correlations between e.s.d.'s in cell parameters are only used when they are defined by crystal symmetry. An approximate (isotropic) treatment of cell e.s.d.'s is used for estimating e.s.d.'s involving l.s. planes.

### Fractional atomic coordinates and isotropic or equivalent isotropic displacement parameters (Å^2^)

**Table:**

	*x*	*y*	*z*	*U*_iso_*/*U*_eq_	
O1	0.6817 (2)	0.38776 (7)	0.3835 (2)	0.0356 (5)	
O2	0.9660 (2)	0.29325 (8)	0.4251 (2)	0.0377 (6)	
O3	0.8861 (2)	0.39337 (7)	0.4678 (2)	0.0365 (6)	
H3	0.8847	0.4219	0.4460	0.055*	
O4	0.9400 (2)	0.29834 (9)	0.6349 (2)	0.0473 (7)	
O5	0.5217 (3)	0.41252 (13)	0.1295 (3)	0.0741 (10)	
C1	0.7673 (3)	0.32247 (10)	0.4929 (3)	0.0295 (7)	
C2	0.7609 (3)	0.37755 (11)	0.4896 (3)	0.0319 (7)	
C3	0.6765 (3)	0.34915 (11)	0.2951 (3)	0.0313 (7)	
H3A	0.5875	0.3370	0.2949	0.038*	
C4	0.7608 (3)	0.31057 (10)	0.3520 (3)	0.0294 (7)	
H4	0.7213	0.2789	0.3393	0.035*	
C5	0.9002 (3)	0.30976 (11)	0.3106 (3)	0.0318 (7)	
H5	0.9277	0.3428	0.2925	0.038*	
C6	0.8988 (3)	0.30454 (10)	0.5302 (3)	0.0317 (8)	
C7	0.6632 (3)	0.30040 (12)	0.5743 (3)	0.0394 (8)	
H7A	0.5807	0.3130	0.5485	0.059*	
H7B	0.6784	0.3079	0.6639	0.059*	
H7C	0.6638	0.2661	0.5628	0.059*	
C8	0.7064 (4)	0.40203 (12)	0.6048 (3)	0.0443 (9)	
H8A	0.7581	0.3946	0.6796	0.066*	
H8B	0.6193	0.3912	0.6188	0.066*	
H8C	0.7064	0.4362	0.5908	0.066*	
C9	0.7099 (3)	0.36289 (12)	0.1567 (3)	0.0374 (8)	
C10	0.5949 (4)	0.38274 (16)	0.0850 (4)	0.0546 (11)	
C11	0.5928 (5)	0.3623 (2)	−0.0398 (4)	0.0716 (14)	
H11	0.5383	0.3718	−0.1069	0.086*	
C12	0.6793 (5)	0.32776 (18)	−0.0477 (4)	0.0645 (12)	
H12	0.6968	0.3110	−0.1239	0.077*	
C13	0.7463 (4)	0.31812 (13)	0.0771 (4)	0.0428 (9)	
H13	0.6996	0.2913	0.1170	0.051*	
C14	0.8858 (4)	0.30276 (13)	0.0714 (4)	0.0446 (9)	
H14	0.9385	0.3315	0.0570	0.054*	
C15	0.9297 (4)	0.27972 (12)	0.1967 (3)	0.0418 (9)	
H15A	0.8875	0.2488	0.2063	0.050*	
H15B	1.0223	0.2742	0.1928	0.050*	
C16	0.8068 (4)	0.40361 (12)	0.1463 (3)	0.0427 (9)	
H16A	0.8258	0.4096	0.0566	0.064*	
H16B	0.8849	0.3949	0.1909	0.064*	
H16C	0.7711	0.4321	0.1848	0.064*	
C17	0.9131 (6)	0.26812 (17)	−0.0367 (4)	0.0723 (15)	
H17A	0.8584	0.2404	−0.0278	0.108*	
H17B	1.0021	0.2583	−0.0330	0.108*	
H17C	0.8964	0.2834	−0.1185	0.108*	
O6	0.2271 (3)	0.47141 (8)	0.6628 (3)	0.0565 (8)	
O7	0.3046 (3)	0.33787 (10)	0.7457 (2)	0.0548 (8)	
O8	0.3753 (3)	0.44016 (11)	0.7977 (3)	0.0672 (9)	
H8	0.4130	0.4633	0.8302	0.101*	
O9	0.2418 (4)	0.34948 (12)	0.9465 (3)	0.0802 (11)	
O10	0.1310 (3)	0.51587 (8)	0.3892 (3)	0.0620 (8)	
C18	0.1688 (4)	0.40306 (13)	0.7805 (4)	0.0442 (9)	
C19	0.2444 (4)	0.45107 (14)	0.7832 (4)	0.0489 (10)	
C20	0.1707 (3)	0.43966 (10)	0.5749 (3)	0.0353 (8)	
H20	0.0799	0.4491	0.5647	0.042*	
C21	0.1730 (3)	0.39150 (11)	0.6398 (3)	0.0330 (7)	
H21	0.0976	0.3723	0.6145	0.040*	
C22	0.2951 (3)	0.36293 (12)	0.6250 (3)	0.0386 (8)	
H22	0.3683	0.3851	0.6171	0.046*	
C23	0.2409 (4)	0.36239 (15)	0.8373 (4)	0.0553 (11)	
C24	0.0352 (4)	0.40600 (16)	0.8354 (4)	0.0561 (11)	
H24A	−0.0105	0.3767	0.8171	0.084*	
H24B	−0.0099	0.4326	0.7967	0.084*	
H24C	0.0400	0.4106	0.9275	0.084*	
C25	0.2053 (5)	0.48671 (17)	0.8816 (4)	0.0726 (14)	
H25A	0.2602	0.5145	0.8755	0.109*	
H25B	0.2133	0.4728	0.9665	0.109*	
H25C	0.1171	0.4961	0.8670	0.109*	
C26	0.2326 (3)	0.44109 (11)	0.4425 (4)	0.0345 (8)	
C27	0.1662 (4)	0.47665 (11)	0.3560 (4)	0.0459 (9)	
C28	0.1556 (5)	0.45658 (14)	0.2310 (4)	0.0567 (11)	
H28	0.1288	0.4731	0.1572	0.068*	
C29	0.1891 (4)	0.41138 (14)	0.2341 (4)	0.0483 (10)	
H29	0.1966	0.3922	0.1605	0.058*	
C30	0.2139 (3)	0.39387 (11)	0.3672 (3)	0.0334 (7)	
H30	0.1319	0.3803	0.3978	0.040*	
C31	0.3146 (3)	0.35456 (11)	0.3859 (3)	0.0350 (8)	
H31	0.4004	0.3695	0.3855	0.042*	
C32	0.2963 (3)	0.32945 (11)	0.5136 (3)	0.0358 (8)	
H32A	0.2152	0.3119	0.5118	0.043*	
H32B	0.3655	0.3063	0.5255	0.043*	
C33	0.3739 (4)	0.45762 (13)	0.4442 (4)	0.0506 (10)	
H33A	0.4113	0.4529	0.3600	0.076*	
H33B	0.4211	0.4392	0.5071	0.076*	
H33C	0.3777	0.4911	0.4667	0.076*	
C34	0.3095 (4)	0.31824 (12)	0.2785 (4)	0.0457 (9)	
H34A	0.3702	0.2929	0.2960	0.069*	
H34B	0.3313	0.3335	0.1979	0.069*	
H34C	0.2239	0.3050	0.2730	0.069*	
O1W	0.5000	0.5000	−0.0109 (5)	0.0797 (14)	
H1W	0.489 (7)	0.4719 (14)	0.043 (5)	0.120*	

### Atomic displacement parameters (Å^2^)

**Table:**

	*U*^11^	*U*^22^	*U*^33^	*U*^12^	*U*^13^	*U*^23^
O1	0.0386 (13)	0.0341 (11)	0.0342 (13)	0.0081 (10)	−0.0026 (11)	0.0019 (10)
O2	0.0333 (13)	0.0367 (13)	0.0433 (14)	0.0071 (10)	−0.0067 (12)	0.0040 (11)
O3	0.0342 (13)	0.0259 (11)	0.0495 (15)	−0.0051 (10)	−0.0021 (11)	0.0033 (11)
O4	0.0521 (16)	0.0495 (15)	0.0403 (15)	−0.0028 (12)	−0.0176 (13)	0.0100 (13)
O5	0.061 (2)	0.104 (3)	0.0569 (18)	0.0353 (19)	−0.0044 (17)	0.0231 (19)
C1	0.0315 (18)	0.0256 (15)	0.0315 (18)	−0.0051 (13)	−0.0032 (14)	0.0032 (14)
C2	0.0333 (17)	0.0312 (16)	0.0313 (18)	0.0017 (14)	−0.0012 (14)	0.0016 (14)
C3	0.0238 (16)	0.0374 (17)	0.0328 (17)	−0.0026 (14)	−0.0020 (15)	0.0028 (14)
C4	0.0303 (17)	0.0271 (15)	0.0309 (17)	−0.0086 (13)	−0.0035 (14)	0.0001 (14)
C5	0.0293 (17)	0.0292 (16)	0.0367 (19)	0.0029 (13)	−0.0033 (14)	0.0056 (14)
C6	0.0363 (19)	0.0234 (15)	0.035 (2)	−0.0034 (14)	−0.0064 (16)	0.0054 (14)
C7	0.0409 (19)	0.0405 (18)	0.0367 (19)	−0.0082 (16)	0.0038 (17)	0.0020 (16)
C8	0.054 (2)	0.0390 (19)	0.040 (2)	0.0013 (17)	0.0047 (18)	−0.0040 (17)
C9	0.0368 (19)	0.0446 (18)	0.0309 (18)	0.0011 (15)	−0.0029 (15)	0.0042 (16)
C10	0.051 (2)	0.071 (3)	0.042 (2)	0.009 (2)	−0.004 (2)	0.019 (2)
C11	0.065 (3)	0.105 (4)	0.045 (3)	0.007 (3)	−0.019 (2)	0.009 (3)
C12	0.079 (3)	0.083 (3)	0.032 (2)	−0.008 (3)	−0.010 (2)	−0.001 (2)
C13	0.051 (2)	0.045 (2)	0.0325 (18)	−0.0086 (17)	−0.0054 (18)	0.0002 (16)
C14	0.058 (2)	0.0407 (19)	0.0349 (19)	0.0012 (18)	0.0070 (19)	−0.0034 (16)
C15	0.046 (2)	0.0361 (19)	0.043 (2)	0.0073 (16)	0.0024 (18)	−0.0056 (17)
C16	0.052 (2)	0.0374 (18)	0.0388 (19)	−0.0001 (16)	0.0101 (18)	0.0084 (16)
C17	0.114 (4)	0.061 (3)	0.042 (2)	0.016 (3)	0.005 (3)	−0.014 (2)
O6	0.087 (2)	0.0373 (13)	0.0453 (16)	−0.0181 (13)	−0.0064 (14)	−0.0092 (12)
O7	0.0617 (19)	0.0587 (17)	0.0439 (16)	0.0192 (15)	−0.0111 (13)	0.0038 (13)
O8	0.0531 (19)	0.0671 (19)	0.082 (2)	−0.0024 (15)	−0.0181 (17)	−0.0196 (17)
O9	0.114 (3)	0.086 (2)	0.0410 (18)	0.022 (2)	−0.0086 (18)	0.0097 (16)
O10	0.070 (2)	0.0259 (13)	0.090 (2)	0.0007 (13)	−0.0007 (18)	0.0017 (13)
C18	0.044 (2)	0.050 (2)	0.038 (2)	0.0066 (18)	−0.0017 (18)	−0.0070 (17)
C19	0.048 (2)	0.048 (2)	0.051 (2)	0.0038 (18)	−0.011 (2)	−0.0092 (19)
C20	0.0372 (18)	0.0252 (15)	0.0434 (19)	−0.0043 (14)	0.0005 (17)	−0.0073 (14)
C21	0.0292 (17)	0.0304 (15)	0.0394 (18)	−0.0022 (14)	0.0021 (15)	−0.0054 (15)
C22	0.0354 (19)	0.0384 (18)	0.042 (2)	0.0021 (15)	−0.0058 (17)	−0.0028 (16)
C23	0.066 (3)	0.059 (2)	0.041 (2)	0.003 (2)	−0.002 (2)	−0.003 (2)
C24	0.056 (3)	0.067 (3)	0.045 (2)	−0.001 (2)	0.011 (2)	−0.003 (2)
C25	0.080 (3)	0.068 (3)	0.070 (3)	−0.002 (3)	0.011 (3)	−0.028 (3)
C26	0.0318 (17)	0.0236 (15)	0.048 (2)	−0.0063 (13)	0.0020 (16)	−0.0044 (15)
C27	0.049 (2)	0.0280 (18)	0.061 (2)	−0.0071 (16)	0.005 (2)	0.0074 (18)
C28	0.073 (3)	0.047 (2)	0.050 (2)	0.005 (2)	0.001 (2)	0.016 (2)
C29	0.055 (2)	0.050 (2)	0.040 (2)	0.001 (2)	0.0006 (19)	0.0001 (17)
C30	0.0336 (18)	0.0268 (15)	0.0399 (18)	−0.0037 (13)	0.0045 (15)	−0.0045 (15)
C31	0.0311 (18)	0.0289 (16)	0.045 (2)	0.0000 (14)	0.0039 (16)	−0.0059 (15)
C32	0.0319 (19)	0.0267 (15)	0.049 (2)	0.0080 (13)	−0.0029 (16)	−0.0054 (16)
C33	0.041 (2)	0.042 (2)	0.069 (3)	−0.0165 (18)	0.011 (2)	−0.0112 (19)
C34	0.050 (2)	0.039 (2)	0.048 (2)	0.0037 (17)	0.004 (2)	−0.0099 (17)
O1W	0.086 (3)	0.081 (3)	0.072 (3)	−0.001 (3)	0.000	0.000

### Geometric parameters (Å, º)

**Table:**

O1—C2	1.424 (4)	O6—C20	1.421 (4)
O1—C3	1.438 (4)	O7—C23	1.365 (5)
O2—C6	1.352 (4)	O7—C22	1.459 (4)
O2—C5	1.468 (4)	O8—C19	1.424 (5)
O3—C2	1.415 (4)	O8—H8	0.8400
O3—H3	0.8400	O9—C23	1.206 (5)
O4—C6	1.198 (4)	O10—C27	1.224 (4)
O5—C10	1.237 (5)	C18—C23	1.506 (6)
C1—C4	1.522 (5)	C18—C21	1.517 (5)
C1—C7	1.527 (5)	C18—C24	1.526 (6)
C1—C6	1.529 (5)	C18—C19	1.578 (6)
C1—C2	1.564 (4)	C19—C25	1.505 (6)
C2—C8	1.511 (5)	C20—C21	1.528 (4)
C3—C4	1.532 (5)	C20—C26	1.540 (5)
C3—C9	1.548 (5)	C20—H20	1.0000
C3—H3A	1.0000	C21—C22	1.530 (5)
C4—C5	1.534 (5)	C21—H21	1.0000
C4—H4	1.0000	C22—C32	1.508 (5)
C5—C15	1.504 (5)	C22—H22	1.0000
C5—H5	1.0000	C24—H24A	0.9800
C7—H7A	0.9800	C24—H24B	0.9800
C7—H7B	0.9800	C24—H24C	0.9800
C7—H7C	0.9800	C25—H25A	0.9800
C8—H8A	0.9800	C25—H25B	0.9800
C8—H8B	0.9800	C25—H25C	0.9800
C8—H8C	0.9800	C26—C27	1.529 (5)
C9—C10	1.535 (5)	C26—C33	1.562 (5)
C9—C16	1.546 (5)	C26—C30	1.569 (4)
C9—C13	1.570 (5)	C27—C28	1.438 (6)
C10—C11	1.437 (6)	C28—C29	1.331 (5)
C11—C12	1.340 (7)	C28—H28	0.9500
C11—H11	0.9500	C29—C30	1.509 (5)
C12—C13	1.516 (6)	C29—H29	0.9500
C12—H12	0.9500	C30—C31	1.552 (5)
C13—C14	1.536 (6)	C30—H30	1.0000
C13—H13	1.0000	C31—C34	1.530 (5)
C14—C17	1.530 (6)	C31—C32	1.534 (5)
C14—C15	1.544 (5)	C31—H31	1.0000
C14—H14	1.0000	C32—H32A	0.9900
C15—H15A	0.9900	C32—H32B	0.9900
C15—H15B	0.9900	C33—H33A	0.9800
C16—H16A	0.9800	C33—H33B	0.9800
C16—H16B	0.9800	C33—H33C	0.9800
C16—H16C	0.9800	C34—H34A	0.9800
C17—H17A	0.9800	C34—H34B	0.9800
C17—H17B	0.9800	C34—H34C	0.9800
C17—H17C	0.9800	O1W—H1W	0.99 (3)
O6—C19	1.404 (5)		
			
C2—O1—C3	112.0 (2)	C19—O6—C20	112.4 (3)
C6—O2—C5	110.3 (2)	C23—O7—C22	109.4 (3)
C2—O3—H3	109.5	C19—O8—H8	109.5
C4—C1—C7	115.0 (3)	C23—C18—C21	101.9 (3)
C4—C1—C6	102.5 (3)	C23—C18—C24	111.0 (4)
C7—C1—C6	111.8 (3)	C21—C18—C24	114.1 (3)
C4—C1—C2	101.4 (3)	C23—C18—C19	113.5 (3)
C7—C1—C2	113.0 (3)	C21—C18—C19	100.9 (3)
C6—C1—C2	112.2 (3)	C24—C18—C19	114.4 (3)
O3—C2—O1	110.9 (3)	O6—C19—O8	108.2 (4)
O3—C2—C8	109.9 (3)	O6—C19—C25	108.0 (3)
O1—C2—C8	108.2 (3)	O8—C19—C25	109.7 (3)
O3—C2—C1	106.3 (2)	O6—C19—C18	105.8 (3)
O1—C2—C1	104.2 (3)	O8—C19—C18	107.7 (3)
C8—C2—C1	117.3 (3)	C25—C19—C18	117.0 (4)
O1—C3—C4	105.6 (2)	O6—C20—C21	105.6 (3)
O1—C3—C9	114.1 (3)	O6—C20—C26	113.2 (3)
C4—C3—C9	114.6 (3)	C21—C20—C26	114.9 (3)
O1—C3—H3A	107.4	O6—C20—H20	107.6
C4—C3—H3A	107.4	C21—C20—H20	107.6
C9—C3—H3A	107.4	C26—C20—H20	107.6
C1—C4—C3	104.4 (3)	C18—C21—C20	104.0 (3)
C1—C4—C5	103.7 (3)	C18—C21—C22	103.8 (3)
C3—C4—C5	117.1 (3)	C20—C21—C22	116.2 (3)
C1—C4—H4	110.4	C18—C21—H21	110.8
C3—C4—H4	110.4	C20—C21—H21	110.8
C5—C4—H4	110.4	C22—C21—H21	110.8
O2—C5—C15	112.0 (3)	O7—C22—C32	111.7 (3)
O2—C5—C4	103.1 (3)	O7—C22—C21	103.2 (3)
C15—C5—C4	115.7 (3)	C32—C22—C21	114.8 (3)
O2—C5—H5	108.6	O7—C22—H22	109.0
C15—C5—H5	108.6	C32—C22—H22	109.0
C4—C5—H5	108.6	C21—C22—H22	109.0
O4—C6—O2	121.8 (3)	O9—C23—O7	120.9 (4)
O4—C6—C1	127.9 (3)	O9—C23—C18	127.9 (4)
O2—C6—C1	110.2 (3)	O7—C23—C18	111.0 (3)
C1—C7—H7A	109.5	C18—C24—H24A	109.5
C1—C7—H7B	109.5	C18—C24—H24B	109.5
H7A—C7—H7B	109.5	H24A—C24—H24B	109.5
C1—C7—H7C	109.5	C18—C24—H24C	109.5
H7A—C7—H7C	109.5	H24A—C24—H24C	109.5
H7B—C7—H7C	109.5	H24B—C24—H24C	109.5
C2—C8—H8A	109.5	C19—C25—H25A	109.5
C2—C8—H8B	109.5	C19—C25—H25B	109.5
H8A—C8—H8B	109.5	H25A—C25—H25B	109.5
C2—C8—H8C	109.5	C19—C25—H25C	109.5
H8A—C8—H8C	109.5	H25A—C25—H25C	109.5
H8B—C8—H8C	109.5	H25B—C25—H25C	109.5
C10—C9—C16	102.3 (3)	C27—C26—C20	111.2 (3)
C10—C9—C3	112.0 (3)	C27—C26—C33	104.2 (3)
C16—C9—C3	113.9 (3)	C20—C26—C33	113.7 (3)
C10—C9—C13	103.2 (3)	C27—C26—C30	101.9 (3)
C16—C9—C13	113.9 (3)	C20—C26—C30	112.4 (3)
C3—C9—C13	110.7 (3)	C33—C26—C30	112.5 (3)
O5—C10—C11	127.8 (4)	O10—C27—C28	126.7 (4)
O5—C10—C9	123.9 (4)	O10—C27—C26	124.7 (4)
C11—C10—C9	108.2 (4)	C28—C27—C26	108.6 (3)
C12—C11—C10	110.0 (4)	C29—C28—C27	109.8 (4)
C12—C11—H11	125.0	C29—C28—H28	125.1
C10—C11—H11	125.0	C27—C28—H28	125.1
C11—C12—C13	113.3 (4)	C28—C29—C30	112.7 (3)
C11—C12—H12	123.4	C28—C29—H29	123.6
C13—C12—H12	123.4	C30—C29—H29	123.6
C12—C13—C14	117.7 (4)	C29—C30—C31	118.2 (3)
C12—C13—C9	101.7 (3)	C29—C30—C26	102.1 (3)
C14—C13—C9	119.0 (3)	C31—C30—C26	117.6 (3)
C12—C13—H13	105.8	C29—C30—H30	106.0
C14—C13—H13	105.8	C31—C30—H30	106.0
C9—C13—H13	105.8	C26—C30—H30	106.0
C17—C14—C13	113.1 (4)	C34—C31—C32	109.3 (3)
C17—C14—C15	107.8 (3)	C34—C31—C30	111.5 (3)
C13—C14—C15	112.0 (3)	C32—C31—C30	111.0 (3)
C17—C14—H14	107.9	C34—C31—H31	108.3
C13—C14—H14	107.9	C32—C31—H31	108.3
C15—C14—H14	107.9	C30—C31—H31	108.3
C5—C15—C14	112.3 (3)	C22—C32—C31	112.9 (3)
C5—C15—H15A	109.2	C22—C32—H32A	109.0
C14—C15—H15A	109.2	C31—C32—H32A	109.0
C5—C15—H15B	109.2	C22—C32—H32B	109.0
C14—C15—H15B	109.2	C31—C32—H32B	109.0
H15A—C15—H15B	107.9	H32A—C32—H32B	107.8
C9—C16—H16A	109.5	C26—C33—H33A	109.5
C9—C16—H16B	109.5	C26—C33—H33B	109.5
H16A—C16—H16B	109.5	H33A—C33—H33B	109.5
C9—C16—H16C	109.5	C26—C33—H33C	109.5
H16A—C16—H16C	109.5	H33A—C33—H33C	109.5
H16B—C16—H16C	109.5	H33B—C33—H33C	109.5
C14—C17—H17A	109.5	C31—C34—H34A	109.5
C14—C17—H17B	109.5	C31—C34—H34B	109.5
H17A—C17—H17B	109.5	H34A—C34—H34B	109.5
C14—C17—H17C	109.5	C31—C34—H34C	109.5
H17A—C17—H17C	109.5	H34A—C34—H34C	109.5
H17B—C17—H17C	109.5	H34B—C34—H34C	109.5
			
C3—O1—C2—O3	92.6 (3)	C20—O6—C19—O8	104.5 (4)
C3—O1—C2—C8	−146.9 (3)	C20—O6—C19—C25	−136.8 (4)
C3—O1—C2—C1	−21.4 (3)	C20—O6—C19—C18	−10.8 (4)
C4—C1—C2—O3	−83.8 (3)	C23—C18—C19—O6	135.6 (3)
C7—C1—C2—O3	152.5 (3)	C21—C18—C19—O6	27.3 (4)
C6—C1—C2—O3	24.9 (4)	C24—C18—C19—O6	−95.6 (4)
C4—C1—C2—O1	33.3 (3)	C23—C18—C19—O8	20.0 (5)
C7—C1—C2—O1	−90.4 (3)	C21—C18—C19—O8	−88.3 (4)
C6—C1—C2—O1	142.1 (3)	C24—C18—C19—O8	148.8 (4)
C4—C1—C2—C8	152.9 (3)	C23—C18—C19—C25	−104.1 (4)
C7—C1—C2—C8	29.2 (4)	C21—C18—C19—C25	147.6 (4)
C6—C1—C2—C8	−98.3 (4)	C24—C18—C19—C25	24.7 (5)
C2—O1—C3—C4	0.3 (3)	C19—O6—C20—C21	−10.5 (4)
C2—O1—C3—C9	−126.4 (3)	C19—O6—C20—C26	−137.1 (3)
C7—C1—C4—C3	89.3 (3)	C23—C18—C21—C20	−149.9 (3)
C6—C1—C4—C3	−149.0 (2)	C24—C18—C21—C20	90.4 (4)
C2—C1—C4—C3	−33.0 (3)	C19—C18—C21—C20	−32.7 (3)
C7—C1—C4—C5	−147.5 (3)	C23—C18—C21—C22	−27.8 (4)
C6—C1—C4—C5	−25.9 (3)	C24—C18—C21—C22	−147.5 (3)
C2—C1—C4—C5	90.2 (3)	C19—C18—C21—C22	89.3 (3)
O1—C3—C4—C1	21.6 (3)	O6—C20—C21—C18	28.0 (4)
C9—C3—C4—C1	148.0 (3)	C26—C20—C21—C18	153.6 (3)
O1—C3—C4—C5	−92.4 (3)	O6—C20—C21—C22	−85.4 (3)
C9—C3—C4—C5	34.0 (4)	C26—C20—C21—C22	40.2 (4)
C6—O2—C5—C15	−151.6 (3)	C23—O7—C22—C32	−148.9 (3)
C6—O2—C5—C4	−26.6 (3)	C23—O7—C22—C21	−25.1 (4)
C1—C4—C5—O2	31.9 (3)	C18—C21—C22—O7	32.6 (3)
C3—C4—C5—O2	146.3 (3)	C20—C21—C22—O7	146.1 (3)
C1—C4—C5—C15	154.5 (3)	C18—C21—C22—C32	154.3 (3)
C3—C4—C5—C15	−91.1 (3)	C20—C21—C22—C32	−92.2 (4)
C5—O2—C6—O4	−173.1 (3)	C22—O7—C23—O9	−176.0 (4)
C5—O2—C6—C1	10.2 (3)	C22—O7—C23—C18	7.4 (5)
C4—C1—C6—O4	−165.8 (3)	C21—C18—C23—O9	−162.7 (5)
C7—C1—C6—O4	−42.0 (4)	C24—C18—C23—O9	−40.8 (6)
C2—C1—C6—O4	86.1 (4)	C19—C18—C23—O9	89.6 (6)
C4—C1—C6—O2	10.7 (3)	C21—C18—C23—O7	13.6 (4)
C7—C1—C6—O2	134.5 (3)	C24—C18—C23—O7	135.5 (4)
C2—C1—C6—O2	−97.3 (3)	C19—C18—C23—O7	−94.1 (4)
O1—C3—C9—C10	−84.3 (4)	O6—C20—C26—C27	−91.2 (4)
C4—C3—C9—C10	153.8 (3)	C21—C20—C26—C27	147.3 (3)
O1—C3—C9—C16	31.2 (4)	O6—C20—C26—C33	26.1 (4)
C4—C3—C9—C16	−90.6 (3)	C21—C20—C26—C33	−95.4 (3)
O1—C3—C9—C13	161.1 (3)	O6—C20—C26—C30	155.3 (3)
C4—C3—C9—C13	39.3 (4)	C21—C20—C26—C30	33.8 (4)
C16—C9—C10—O5	−76.5 (5)	C20—C26—C27—O10	42.1 (5)
C3—C9—C10—O5	45.9 (5)	C33—C26—C27—O10	−80.8 (4)
C13—C9—C10—O5	165.1 (4)	C30—C26—C27—O10	162.0 (4)
C16—C9—C10—C11	101.2 (4)	C20—C26—C27—C28	−139.2 (3)
C3—C9—C10—C11	−136.4 (4)	C33—C26—C27—C28	97.8 (4)
C13—C9—C10—C11	−17.2 (4)	C30—C26—C27—C28	−19.3 (4)
O5—C10—C11—C12	−173.5 (5)	O10—C27—C28—C29	−172.2 (4)
C9—C10—C11—C12	8.9 (5)	C26—C27—C28—C29	9.2 (5)
C10—C11—C12—C13	4.0 (6)	C27—C28—C29—C30	6.1 (5)
C11—C12—C13—C14	−146.4 (4)	C28—C29—C30—C31	−148.7 (4)
C11—C12—C13—C9	−14.6 (5)	C28—C29—C30—C26	−17.9 (5)
C10—C9—C13—C12	18.2 (4)	C27—C26—C30—C29	21.2 (4)
C16—C9—C13—C12	−91.9 (4)	C20—C26—C30—C29	140.3 (3)
C3—C9—C13—C12	138.2 (3)	C33—C26—C30—C29	−89.9 (4)
C10—C9—C13—C14	149.2 (3)	C27—C26—C30—C31	152.3 (3)
C16—C9—C13—C14	39.1 (4)	C20—C26—C30—C31	−88.6 (4)
C3—C9—C13—C14	−90.8 (4)	C33—C26—C30—C31	41.3 (4)
C12—C13—C14—C17	−40.3 (5)	C29—C30—C31—C34	−38.4 (4)
C9—C13—C14—C17	−163.8 (3)	C26—C30—C31—C34	−161.6 (3)
C12—C13—C14—C15	−162.4 (3)	C29—C30—C31—C32	−160.4 (3)
C9—C13—C14—C15	74.1 (4)	C26—C30—C31—C32	76.3 (4)
O2—C5—C15—C14	−167.3 (3)	O7—C22—C32—C31	−167.9 (3)
C4—C5—C15—C14	75.0 (4)	C21—C22—C32—C31	75.2 (4)
C17—C14—C15—C5	−176.7 (4)	C34—C31—C32—C22	−177.9 (3)
C13—C14—C15—C5	−51.6 (4)	C30—C31—C32—C22	−54.5 (4)

### Hydrogen-bond geometry (Å, º)

**Table:**

*D*—H···*A*	*D*—H	H···*A*	*D*···*A*	*D*—H···*A*
O3—H3···O10^i^	0.84	1.87	2.711 (3)	175
O8—H8···O1*W*^ii^	0.84	2.17	2.945 (5)	153
O1*W*—H1*W*···O5	0.99 (3)	1.94 (3)	2.897 (4)	162 (6)

Symmetry codes: (i) −*x*+1, −*y*+1, *z*; (ii) *x*, *y*, *z*+1.
